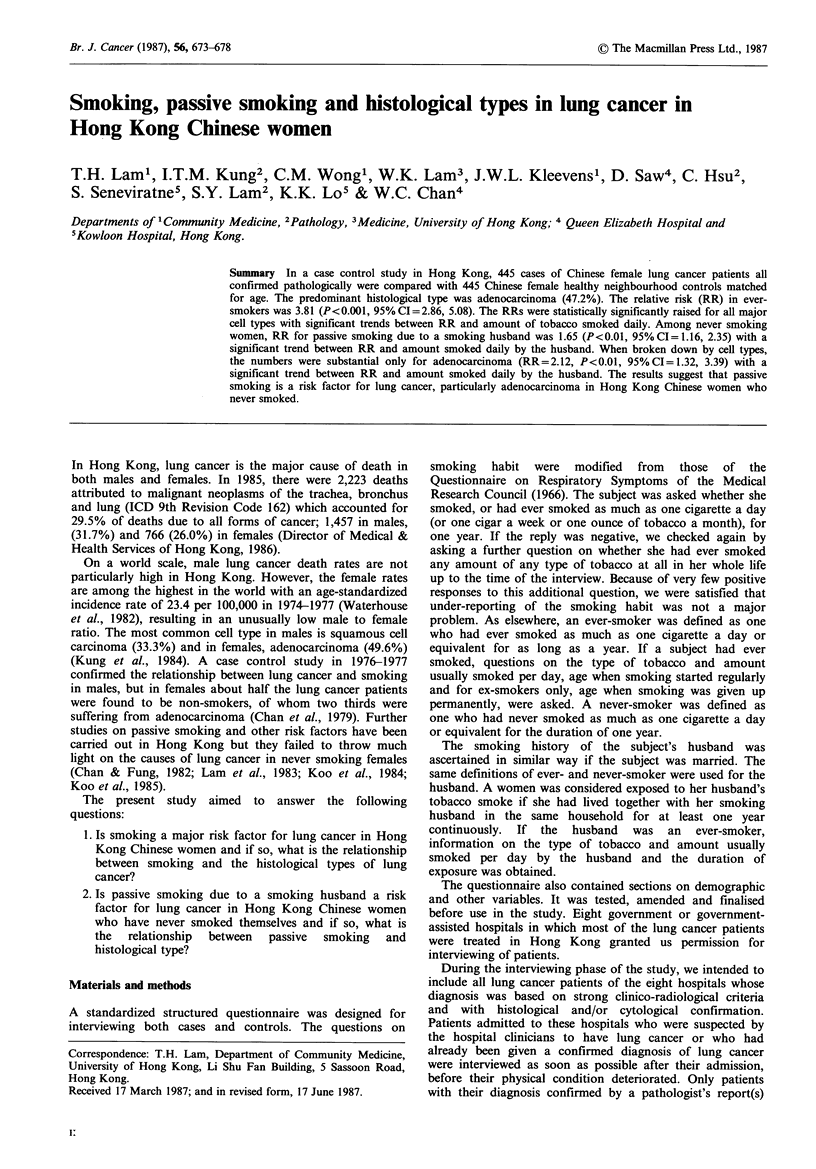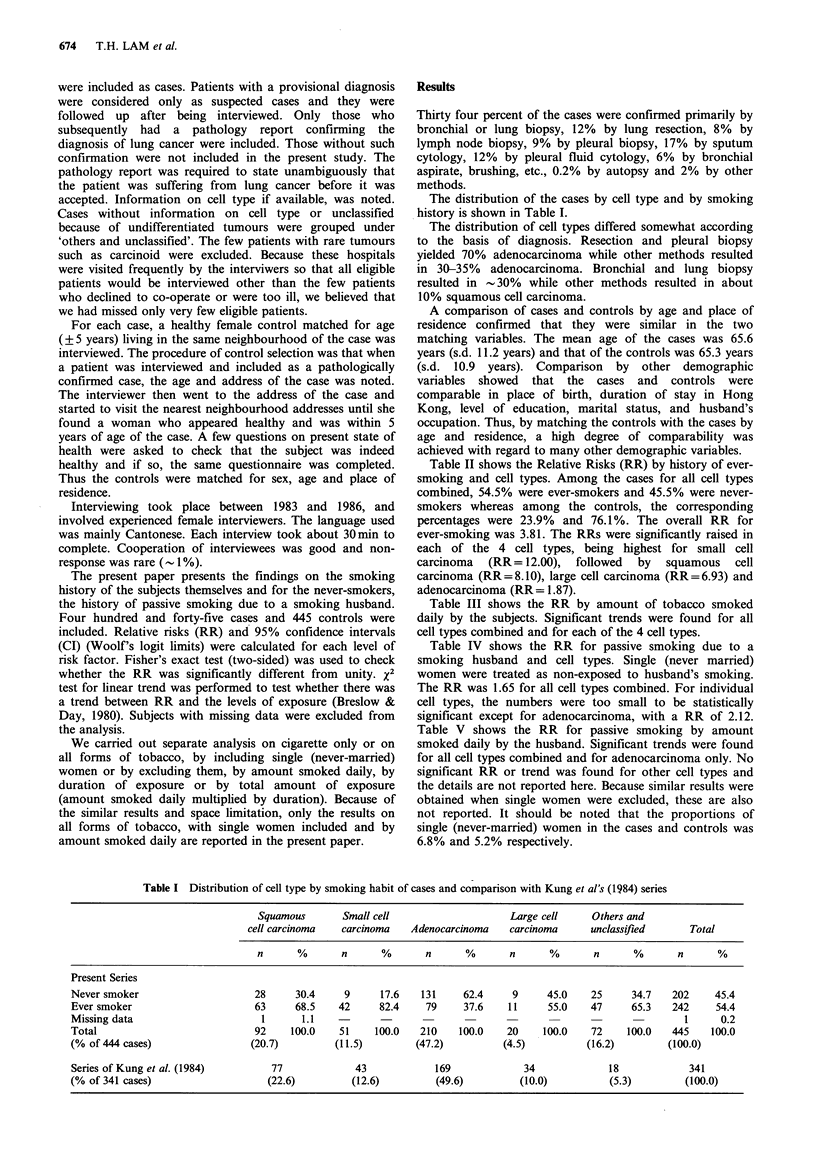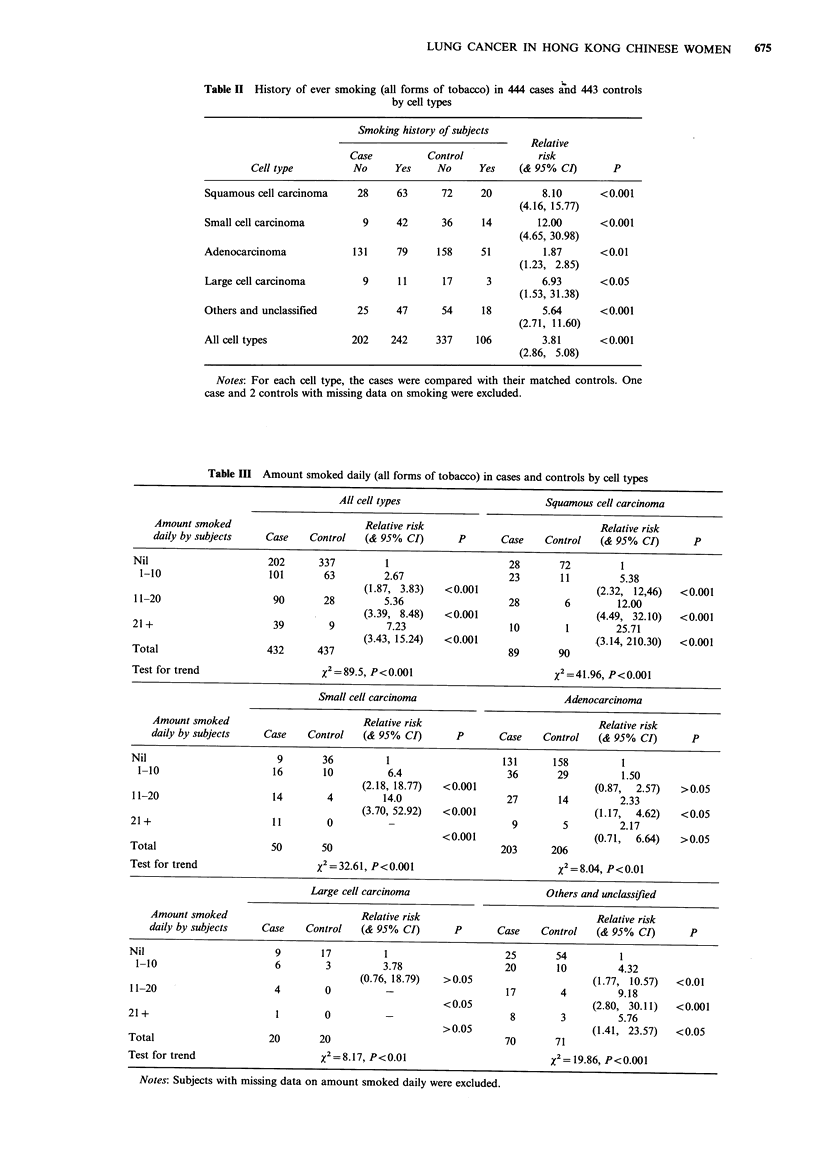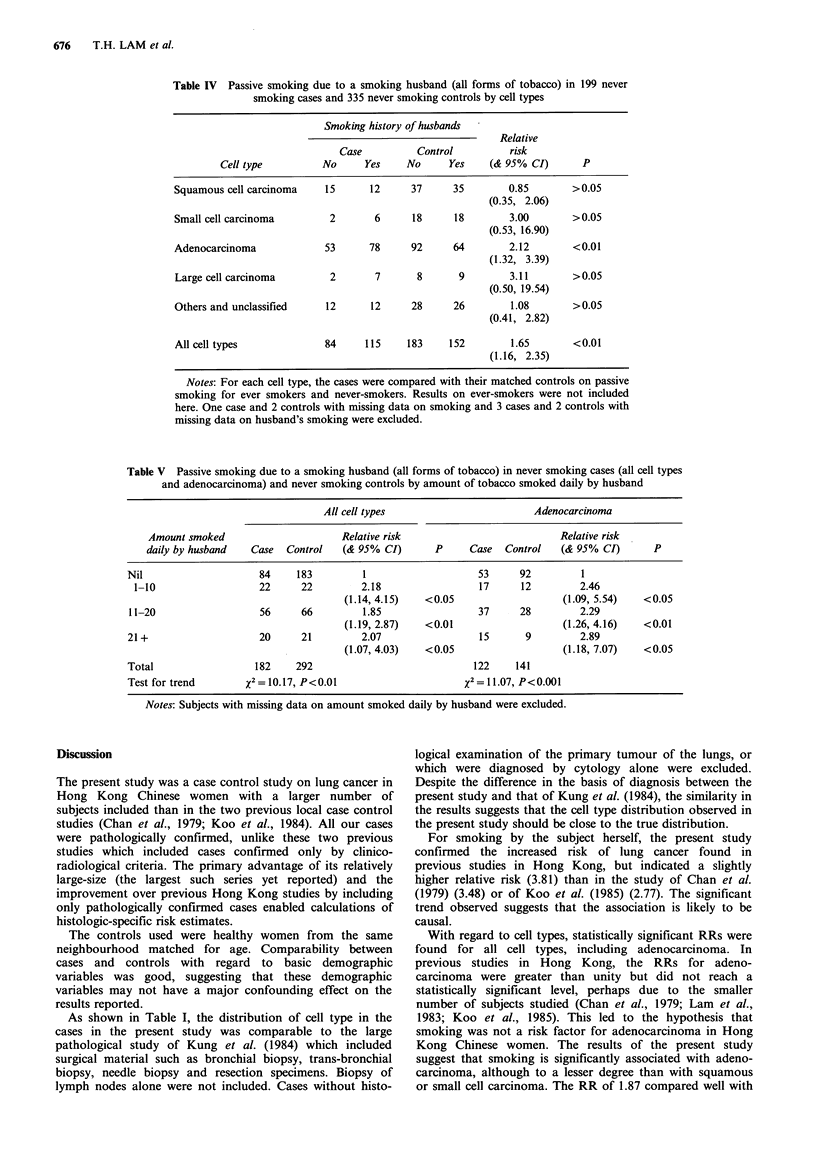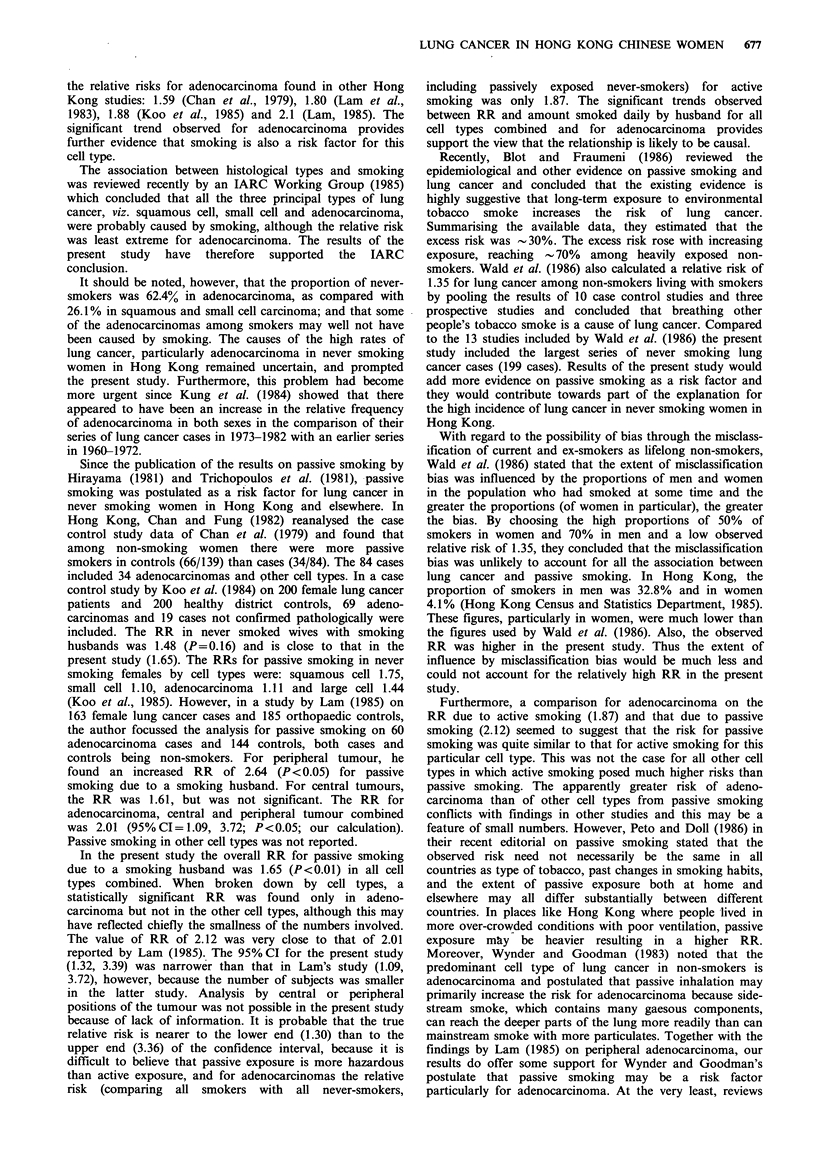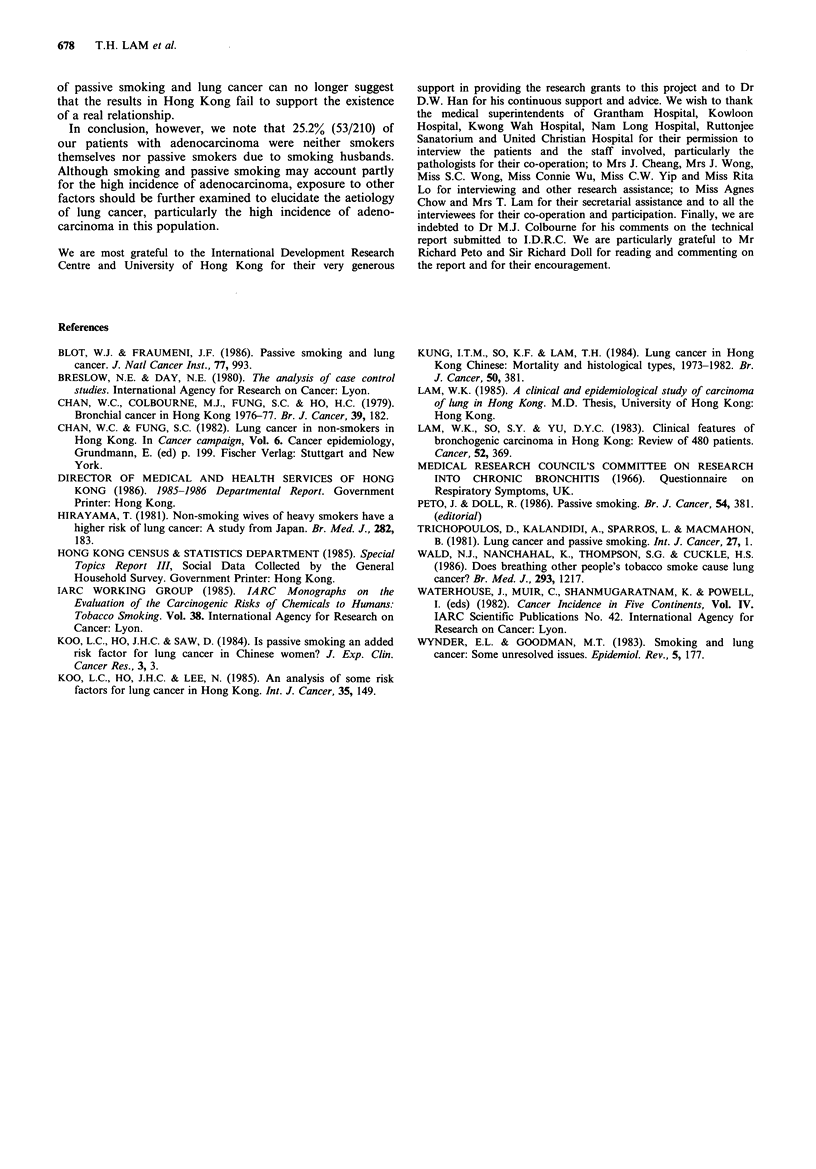# Smoking, passive smoking and histological types in lung cancer in Hong Kong Chinese women.

**DOI:** 10.1038/bjc.1987.264

**Published:** 1987-11

**Authors:** T. H. Lam, I. T. Kung, C. M. Wong, W. K. Lam, J. W. Kleevens, D. Saw, C. Hsu, S. Seneviratne, S. Y. Lam, K. K. Lo

**Affiliations:** Department of Community Medicine, University of Hong Kong.

## Abstract

In a case control study in Hong Kong, 445 cases of Chinese female lung cancer patients all confirmed pathologically were compared with 445 Chinese female healthy neighbourhood controls matched for age. The predominant histological type was adenocarcinoma (47.2%). The relative risk (RR) in ever-smokers was 3.81 (P less than 0.001, 95% CI = 2.86, 5.08). The RRs were statistically significantly raised for all major cell types with significant trends between RR and amount of tobacco smoked daily. Among never smoking women, RR for passive smoking due to a smoking husband was 1.65 (P less than 0.01, 95% CI = 1.16, 2.35) with a significant trend between RR and amount smoked daily by the husband. When broken down by cell types, the numbers were substantial only for adenocarcinoma (RR = 2.12, P less than 0.01, 95% CI = 1.32, 3.39) with a significant trend between RR and amount smoked daily by the husband. The results suggest that passive smoking is a risk factor for lung cancer, particularly adenocarcinoma in Hong Kong Chinese women who never smoked.


Br. J. Cancer (1987), 56, 673–678 © The Macmillan Press Ltd., 1987

### Smoking, passive smoking and histological types in lung cancer in Hong Kong Chinese women

T.H. Lam[1], I.T.M. Kung[2], C.M. Wong[1], W.K. Lam[3], J.W.L. Kleevens[1], D. Saw[4], C. Hsu[2], S. Seneviratne[5], S.Y. Lam[2], K.K. Lo[5] & W.C. Chan[4]

*Departments of [1]Community Medicine, [2]Pathology, [3]Medicine, University of Hong Kong; [4] Queen Elizabeth Hospital and [5]Kowloon Hospital, Hong Kong.*

**Summary** In a case control study in Hong Kong, 445 cases of Chinese female lung cancer patients all confirmed pathologically were compared with 445 Chinese female healthy neighbourhood controls matched for age. The predominant histological type was adenocarcinoma (47.2%). The relative risk (RR) in ever-smokers was 3.81 ($P<0.001$, 95% CI = 2.86, 5.08). The RRs were statistically significantly raised for all major cell types with significant trends between RR and amount of tobacco smoked daily. Among never smoking women, RR for passive smoking due to a smoking husband was 1.65 ($P<0.01$, 95% CI = 1.16, 2.35) with a significant trend between RR and amount smoked daily by the husband. When broken down by cell types, the numbers were substantial only for adenocarcinoma (RR = 2.12, $P<0.01$, 95% CI = 1.32, 3.39) with a significant trend between RR and amount smoked daily by the husband. The results suggest that passive smoking is a risk factor for lung cancer, particularly adenocarcinoma in Hong Kong Chinese women who never smoked.

In Hong Kong, lung cancer is the major cause of death in both males and females. In 1985, there were 2,223 deaths attributed to malignant neoplasms of the trachea, bronchus and lung (ICD 9th Revision Code 162) which accounted for 29.5% of deaths due to all forms of cancer; 1,457 in males, (31.7%) and 766 (26.0%) in females (Director of Medical & Health Services of Hong Kong, 1986).

On a world scale, male lung cancer death rates are not particularly high in Hong Kong. However, the female rates are among the highest in the world with an age-standardized incidence rate of 23.4 per 100,000 in 1974–1977 (Waterhouse *et al.*, 1982), resulting in an unusually low male to female ratio. The most common cell type in males is squamous cell carcinoma (33.3%) and in females, adenocarcinoma (49.6%) (Kung *et al.*, 1984). A case control study in 1976–1977 confirmed the relationship between lung cancer and smoking in males, but in females about half the lung cancer patients were found to be non-smokers, of whom two thirds were suffering from adenocarcinoma (Chan *et al.*, 1979). Further studies on passive smoking and other risk factors have been carried out in Hong Kong but they failed to throw much light on the causes of lung cancer in never smoking females (Chan & Fung, 1982; Lam *et al.*, 1983; Koo *et al.*, 1984; Koo *et al.*, 1985).

The present study aimed to answer the following questions:

1. Is smoking a major risk factor for lung cancer in Hong Kong Chinese women and if so, what is the relationship between smoking and the histological types of lung cancer?
2. Is passive smoking due to a smoking husband a risk factor for lung cancer in Hong Kong Chinese women who have never smoked themselves and if so, what is the relationship between passive smoking and histological type?

### Materials and methods

A standardized structured questionnaire was designed for interviewing both cases and controls. The questions on smoking habit were modified from those of the Questionnaire on Respiratory Symptoms of the Medical Research Council (1966). The subject was asked whether she smoked, or had ever smoked as much as one cigarette a day (or one cigar a week or one ounce of tobacco a month), for one year. If the reply was negative, we checked again by asking a further question on whether she had ever smoked any amount of any type of tobacco at all in her whole life up to the time of the interview. Because of very few positive responses to this additional question, we were satisfied that under-reporting of the smoking habit was not a major problem. As elsewhere, an ever-smoker was defined as one who had ever smoked as much as one cigarette a day or equivalent for as long as a year. If a subject had ever smoked, questions on the type of tobacco and amount usually smoked per day, age when smoking started regularly and for ex-smokers only, age when smoking was given up permanently, were asked. A never-smoker was defined as one who had never smoked as much as one cigarette a day or equivalent for the duration of one year.

The smoking history of the subject's husband was ascertained in similar way if the subject was married. The same definitions of ever- and never-smoker were used for the husband. A women was considered exposed to her husband's tobacco smoke if she had lived together with her smoking husband in the same household for at least one year continuously. If the husband was an ever-smoker, information on the type of tobacco and amount usually smoked per day by the husband and the duration of exposure was obtained.

The questionnaire also contained sections on demographic and other variables. It was tested, amended and finalised before use in the study. Eight government or government-assisted hospitals in which most of the lung cancer patients were treated in Hong Kong granted us permission for interviewing of patients.

During the interviewing phase of the study, we intended to include all lung cancer patients of the eight hospitals whose diagnosis was based on strong clinico-radiological criteria and with histological and/or cytological confirmation. Patients admitted to these hospitals who were suspected by the hospital clinicians to have lung cancer or who had already been given a confirmed diagnosis of lung cancer were interviewed as soon as possible after their admission, before their physical condition deteriorated. Only patients with their diagnosis confirmed by a pathologist's report(s)

Correspondence: T.H. Lam, Department of Community Medicine, University of Hong Kong, Li Shu Fan Building, 5 Sassoon Road, Hong Kong.
Received 17 March 1987; and in revised form, 17 June 1987.

were included as cases. Patients with a provisional diagnosis were considered only as suspected cases and they were followed up after being interviewed. Only those who subsequently had a pathology report confirming the diagnosis of lung cancer were included. Those without such confirmation were not included in the present study. The pathology report was required to state unambiguously that the patient was suffering from lung cancer before it was accepted. Information on cell type if available, was noted. Cases without information on cell type or unclassified because of undifferentiated tumours were grouped under 'others and unclassified'. The few patients with rare tumours such as carcinoid were excluded. Because these hospitals were visited frequently by the interviwers so that all eligible patients would be interviewed other than the few patients who declined to co-operate or were too ill, we believed that we had missed only very few eligible patients.

For each case, a healthy female control matched for age (±5 years) living in the same neighbourhood of the case was interviewed. The procedure of control selection was that when a patient was interviewed and included as a pathologically confirmed case, the age and address of the case was noted. The interviewer then went to the address of the case and started to visit the nearest neighbourhood addresses until she found a woman who appeared healthy and was within 5 years of age of the case. A few questions on present state of health were asked to check that the subject was indeed healthy and if so, the same questionnaire was completed. Thus the controls were matched for sex, age and place of residence.

Interviewing took place between 1983 and 1986, and involved experienced female interviewers. The language used was mainly Cantonese. Each interview took about 30 min to complete. Cooperation of interviewees was good and non-response was rare (~1%).

The present paper presents the findings on the smoking history of the subjects themselves and for the never-smokers, the history of passive smoking due to a smoking husband. Four hundred and forty-five cases and 445 controls were included. Relative risks (RR) and 95% confidence intervals (CI) (Woolf's logit limits) were calculated for each level of risk factor. Fisher's exact test (two-sided) was used to check whether the RR was significantly different from unity. $\chi^2$ test for linear trend was performed to test whether there was a trend between RR and the levels of exposure (Breslow & Day, 1980). Subjects with missing data were excluded from the analysis.

We carried out separate analysis on cigarette only or on all forms of tobacco, by including single (never-married) women or by excluding them, by amount smoked daily, by duration of exposure or by total amount of exposure (amount smoked daily multiplied by duration). Because of the similar results and space limitation, only the results on all forms of tobacco, with single women included and by amount smoked daily are reported in the present paper.

### Results

Thirty four percent of the cases were confirmed primarily by bronchial or lung biopsy, 12% by lung resection, 8% by lymph node biopsy, 9% by pleural biopsy, 17% by sputum cytology, 12% by pleural fluid cytology, 6% by bronchial aspirate, brushing, etc., 0.2% by autopsy and 2% by other methods.

The distribution of the cases by cell type and by smoking history is shown in Table I.

The distribution of cell types differed somewhat according to the basis of diagnosis. Resection and pleural biopsy yielded 70% adenocarcinoma while other methods resulted in 30–35% adenocarcinoma. Bronchial and lung biopsy resulted in ~30% while other methods resulted in about 10% squamous cell carcinoma.

A comparison of cases and controls by age and place of residence confirmed that they were similar in the two matching variables. The mean age of the cases was 65.6 years (s.d. 11.2 years) and that of the controls was 65.3 years (s.d. 10.9 years). Comparison by other demographic variables showed that the cases and controls were comparable in place of birth, duration of stay in Hong Kong, level of education, marital status, and husband's occupation. Thus, by matching the controls with the cases by age and residence, a high degree of comparability was achieved with regard to many other demographic variables.

Table II shows the Relative Risks (RR) by history of ever-smoking and cell types. Among the cases for all cell types combined, 54.5% were ever-smokers and 45.5% were never-smokers whereas among the controls, the corresponding percentages were 23.9% and 76.1%. The overall RR for ever-smoking was 3.81. The RRs were significantly raised in each of the 4 cell types, being highest for small cell carcinoma (RR=12.00), followed by squamous cell carcinoma (RR=8.10), large cell carcinoma (RR=6.93) and adenocarcinoma (RR=1.87).

Table III shows the RR by amount of tobacco smoked daily by the subjects. Significant trends were found for all cell types combined and for each of the 4 cell types.

Table IV shows the RR for passive smoking due to a smoking husband and cell types. Single (never married) women were treated as non-exposed to husband's smoking. The RR was 1.65 for all cell types combined. For individual cell types, the numbers were too small to be statistically significant except for adenocarcinoma, with a RR of 2.12. Table V shows the RR for passive smoking by amount smoked daily by the husband. Significant trends were found for all cell types combined and for adenocarcinoma only. No significant RR or trend was found for other cell types and the details are not reported here. Because similar results were obtained when single women were excluded, these are also not reported. It should be noted that the proportions of single (never-married) women in the cases and controls was 6.8% and 5.2% respectively.

**Table I** Distribution of cell type by smoking habit of cases and comparison with Kung *et al's* (1984) series

| | *Squamous cell carcinoma* | | *Small cell carcinoma* | | *Adenocarcinoma* | | *Large cell carcinoma* | | *Others and unclassified* | | *Total* | |
|---|---|---|---|---|---|---|---|---|---|---|---|---|
| | *n* | % | *n* | % | *n* | % | *n* | % | *n* | % | *n* | % |
| Present Series | | | | | | | | | | | | |
| Never smoker | 28 | 30.4 | 9 | 17.6 | 131 | 62.4 | 9 | 45.0 | 25 | 34.7 | 202 | 45.4 |
| Ever smoker | 63 | 68.5 | 42 | 82.4 | 79 | 37.6 | 11 | 55.0 | 47 | 65.3 | 242 | 54.4 |
| Missing data | 1 | 1.1 | — | — | — | — | — | — | — | — | 1 | 0.2 |
| Total | 92 | 100.0 | 51 | 100.0 | 210 | 100.0 | 20 | 100.0 | 72 | 100.0 | 445 | 100.0 |
| (% of 444 cases) | (20.7) | | (11.5) | | (47.2) | | (4.5) | | (16.2) | | (100.0) | |
| Series of Kung *et al.* (1984) | 77 | | 43 | | 169 | | 34 | | 18 | | 341 | |
| (% of 341 cases) | (22.6) | | (12.6) | | (49.6) | | (10.0) | | (5.3) | | (100.0) | |

**Table II** History of ever smoking (all forms of tobacco) in 444 cases and 443 controls by cell types

| Cell type | Smoking history of subjects: Case No | Case Yes | Control No | Control Yes | Relative risk (& 95% CI) | P |
|---|---|---|---|---|---|---|
| Squamous cell carcinoma | 28 | 63 | 72 | 20 | 8.10 (4.16, 15.77) | <0.001 |
| Small cell carcinoma | 9 | 42 | 36 | 14 | 12.00 (4.65, 30.98) | <0.001 |
| Adenocarcinoma | 131 | 79 | 158 | 51 | 1.87 (1.23, 2.85) | <0.01 |
| Large cell carcinoma | 9 | 11 | 17 | 3 | 6.93 (1.53, 31.38) | <0.05 |
| Others and unclassified | 25 | 47 | 54 | 18 | 5.64 (2.71, 11.60) | <0.001 |
| All cell types | 202 | 242 | 337 | 106 | 3.81 (2.86, 5.08) | <0.001 |

*Notes*: For each cell type, the cases were compared with their matched controls. One case and 2 controls with missing data on smoking were excluded.

**Table III** Amount smoked daily (all forms of tobacco) in cases and controls by cell types

| Amount smoked daily by subjects | All cell types: Case | Control | Relative risk (& 95% CI) | P | Squamous cell carcinoma: Case | Control | Relative risk (& 95% CI) | P |
|---|---|---|---|---|---|---|---|---|
| Nil | 202 | 337 | 1 | | 28 | 72 | 1 | |
| 1–10 | 101 | 63 | 2.67 (1.87, 3.83) | <0.001 | 23 | 11 | 5.38 (2.32, 12,46) | <0.001 |
| 11–20 | 90 | 28 | 5.36 (3.39, 8.48) | <0.001 | 28 | 6 | 12.00 (4.49, 32.10) | <0.001 |
| 21+ | 39 | 9 | 7.23 (3.43, 15.24) | <0.001 | 10 | 1 | 25.71 (3.14, 210.30) | <0.001 |
| Total | 432 | 437 | | | 89 | 90 | | |
| Test for trend | $\chi^2=89.5$, $P<0.001$ | | | | $\chi^2=41.96$, $P<0.001$ | | | |

| Amount smoked daily by subjects | Small cell carcinoma: Case | Control | Relative risk (& 95% CI) | P | Adenocarcinoma: Case | Control | Relative risk (& 95% CI) | P |
|---|---|---|---|---|---|---|---|---|
| Nil | 9 | 36 | 1 | | 131 | 158 | 1 | |
| 1–10 | 16 | 10 | 6.4 (2.18, 18.77) | <0.001 | 36 | 29 | 1.50 (0.87, 2.57) | >0.05 |
| 11–20 | 14 | 4 | 14.0 (3.70, 52.92) | <0.001 | 27 | 14 | 2.33 (1.17, 4.62) | <0.05 |
| 21+ | 11 | 0 | – | <0.001 | 9 | 5 | 2.17 (0.71, 6.64) | >0.05 |
| Total | 50 | 50 | | | 203 | 206 | | |
| Test for trend | $\chi^2=32.61$, $P<0.001$ | | | | $\chi^2=8.04$, $P<0.01$ | | | |

| Amount smoked daily by subjects | Large cell carcinoma: Case | Control | Relative risk (& 95% CI) | P | Others and unclassified: Case | Control | Relative risk (& 95% CI) | P |
|---|---|---|---|---|---|---|---|---|
| Nil | 9 | 17 | 1 | | 25 | 54 | 1 | |
| 1–10 | 6 | 3 | 3.78 (0.76, 18.79) | >0.05 | 20 | 10 | 4.32 (1.77, 10.57) | <0.01 |
| 11–20 | 4 | 0 | – | <0.05 | 17 | 4 | 9.18 (2.80, 30.11) | <0.001 |
| 21+ | 1 | 0 | – | >0.05 | 8 | 3 | 5.76 (1.41, 23.57) | <0.05 |
| Total | 20 | 20 | | | 70 | 71 | | |
| Test for trend | $\chi^2=8.17$, $P<0.01$ | | | | $\chi^2=19.86$, $P<0.001$ | | | |

*Notes*: Subjects with missing data on amount smoked daily were excluded.

**Table IV** Passive smoking due to a smoking husband (all forms of tobacco) in 199 never smoking cases and 335 never smoking controls by cell types

| | *Smoking history of husbands* | | | | | |
|---|---|---|---|---|---|---|
| | *Case* | | *Control* | | *Relative risk* | |
| *Cell type* | *No* | *Yes* | *No* | *Yes* | *(& 95% CI)* | *P* |
| Squamous cell carcinoma | 15 | 12 | 37 | 35 | 0.85 (0.35, 2.06) | >0.05 |
| Small cell carcinoma | 2 | 6 | 18 | 18 | 3.00 (0.53, 16.90) | >0.05 |
| Adenocarcinoma | 53 | 78 | 92 | 64 | 2.12 (1.32, 3.39) | <0.01 |
| Large cell carcinoma | 2 | 7 | 8 | 9 | 3.11 (0.50, 19.54) | >0.05 |
| Others and unclassified | 12 | 12 | 28 | 26 | 1.08 (0.41, 2.82) | >0.05 |
| All cell types | 84 | 115 | 183 | 152 | 1.65 (1.16, 2.35) | <0.01 |

*Notes*: For each cell type, the cases were compared with their matched controls on passive smoking for ever smokers and never-smokers. Results on ever-smokers were not included here. One case and 2 controls with missing data on smoking and 3 cases and 2 controls with missing data on husband's smoking were excluded.

**Table V** Passive smoking due to a smoking husband (all forms of tobacco) in never smoking cases (all cell types and adenocarcinoma) and never smoking controls by amount of tobacco smoked daily by husband

| | *All cell types* | | | | *Adenocarcinoma* | | | |
|---|---|---|---|---|---|---|---|---|
| *Amount smoked daily by husband* | *Case* | *Control* | *Relative risk (& 95% CI)* | *P* | *Case* | *Control* | *Relative risk (& 95% CI)* | *P* |
| Nil | 84 | 183 | 1 | | 53 | 92 | 1 | |
| 1–10 | 22 | 22 | 2.18 (1.14, 4.15) | <0.05 | 17 | 12 | 2.46 (1.09, 5.54) | <0.05 |
| 11–20 | 56 | 66 | 1.85 (1.19, 2.87) | <0.01 | 37 | 28 | 2.29 (1.26, 4.16) | <0.01 |
| 21+ | 20 | 21 | 2.07 (1.07, 4.03) | <0.05 | 15 | 9 | 2.89 (1.18, 7.07) | <0.05 |
| Total | 182 | 292 | | | 122 | 141 | | |
| Test for trend | $\chi^2 = 10.17$, $P<0.01$ | | | | $\chi^2 = 11.07$, $P<0.001$ | | | |

*Notes*: Subjects with missing data on amount smoked daily by husband were excluded.

### Discussion

The present study was a case control study on lung cancer in Hong Kong Chinese women with a larger number of subjects included than in the two previous local case control studies (Chan *et al.*, 1979; Koo *et al.*, 1984). All our cases were pathologically confirmed, unlike these two previous studies which included cases confirmed only by clinico-radiological criteria. The primary advantage of its relatively large-size (the largest such series yet reported) and the improvement over previous Hong Kong studies by including only pathologically confirmed cases enabled calculations of histologic-specific risk estimates.

The controls used were healthy women from the same neighbourhood matched for age. Comparability between cases and controls with regard to basic demographic variables was good, suggesting that these demographic variables may not have a major confounding effect on the results reported.

As shown in Table I, the distribution of cell type in the cases in the present study was comparable to the large pathological study of Kung *et al.* (1984) which included surgical material such as bronchial biopsy, trans-bronchial biopsy, needle biopsy and resection specimens. Biopsy of lymph nodes alone were not included. Cases without histological examination of the primary tumour of the lungs, or which were diagnosed by cytology alone were excluded. Despite the difference in the basis of diagnosis between the present study and that of Kung *et al.* (1984), the similarity in the results suggests that the cell type distribution observed in the present study should be close to the true distribution.

For smoking by the subject herself, the present study confirmed the increased risk of lung cancer found in previous studies in Hong Kong, but indicated a slightly higher relative risk (3.81) than in the study of Chan *et al.* (1979) (3.48) or of Koo *et al.* (1985) (2.77). The significant trend observed suggests that the association is likely to be causal.

With regard to cell types, statistically significant RRs were found for all cell types, including adenocarcinoma. In previous studies in Hong Kong, the RRs for adenocarcinoma were greater than unity but did not reach a statistically significant level, perhaps due to the smaller number of subjects studied (Chan *et al.*, 1979; Lam *et al.*, 1983; Koo *et al.*, 1985). This led to the hypothesis that smoking was not a risk factor for adenocarcinoma in Hong Kong Chinese women. The results of the present study suggest that smoking is significantly associated with adenocarcinoma, although to a lesser degree than with squamous or small cell carcinoma. The RR of 1.87 compared well with the relative risks for adenocarcinoma found in other Hong Kong studies: 1.59 (Chan *et al.*, 1979), 1.80 (Lam *et al.*, 1983), 1.88 (Koo *et al.*, 1985) and 2.1 (Lam, 1985). The significant trend observed for adenocarcinoma provides further evidence that smoking is also a risk factor for this cell type.

The association between histological types and smoking was reviewed recently by an IARC Working Group (1985) which concluded that all the three principal types of lung cancer, *viz.* squamous cell, small cell and adenocarcinoma, were probably caused by smoking, although the relative risk was least extreme for adenocarcinoma. The results of the present study have therefore supported the IARC conclusion.

It should be noted, however, that the proportion of never-smokers was 62.4% in adenocarcinoma, as compared with 26.1% in squamous and small cell carcinoma; and that some of the adenocarcinomas among smokers may well not have been caused by smoking. The causes of the high rates of lung cancer, particularly adenocarcinoma in never smoking women in Hong Kong remained uncertain, and prompted the present study. Furthermore, this problem had become more urgent since Kung *et al.* (1984) showed that there appeared to have been an increase in the relative frequency of adenocarcinoma in both sexes in the comparison of their series of lung cancer cases in 1973–1982 with an earlier series in 1960–1972.

Since the publication of the results on passive smoking by Hirayama (1981) and Trichopoulos *et al.* (1981), passive smoking was postulated as a risk factor for lung cancer in never smoking women in Hong Kong and elsewhere. In Hong Kong, Chan and Fung (1982) reanalysed the case control study data of Chan *et al.* (1979) and found that among non-smoking women there were more passive smokers in controls (66/139) than cases (34/84). The 84 cases included 34 adenocarcinomas and other cell types. In a case control study by Koo *et al.* (1984) on 200 female lung cancer patients and 200 healthy district controls, 69 adenocarcinomas and 19 cases not confirmed pathologically were included. The RR in never smoked wives with smoking husbands was 1.48 ($P=0.16$) and is close to that in the present study (1.65). The RRs for passive smoking in never smoking females by cell types were: squamous cell 1.75, small cell 1.10, adenocarcinoma 1.11 and large cell 1.44 (Koo *et al.*, 1985). However, in a study by Lam (1985) on 163 female lung cancer cases and 185 orthopaedic controls, the author focussed the analysis for passive smoking on 60 adenocarcinoma cases and 144 controls, both cases and controls being non-smokers. For peripheral tumour, he found an increased RR of 2.64 ($P<0.05$) for passive smoking due to a smoking husband. For central tumours, the RR was 1.61, but was not significant. The RR for adenocarcinoma, central and peripheral tumour combined was 2.01 (95% CI = 1.09, 3.72; $P<0.05$; our calculation). Passive smoking in other cell types was not reported.

In the present study the overall RR for passive smoking due to a smoking husband was 1.65 ($P<0.01$) in all cell types combined. When broken down by cell types, a statistically significant RR was found only in adenocarcinoma but not in the other cell types, although this may have reflected chiefly the smallness of the numbers involved. The value of RR of 2.12 was very close to that of 2.01 reported by Lam (1985). The 95% CI for the present study (1.32, 3.39) was narrower than that in Lam's study (1.09, 3.72), however, because the number of subjects was smaller in the latter study. Analysis by central or peripheral positions of the tumour was not possible in the present study because of lack of information. It is probable that the true relative risk is nearer to the lower end (1.30) than to the upper end (3.36) of the confidence interval, because it is difficult to believe that passive exposure is more hazardous than active exposure, and for adenocarcinomas the relative risk (comparing all smokers with all never-smokers, including passively exposed never-smokers) for active smoking was only 1.87. The significant trends observed between RR and amount smoked daily by husband for all cell types combined and for adenocarcinoma provides support the view that the relationship is likely to be causal.

Recently, Blot and Fraumeni (1986) reviewed the epidemiological and other evidence on passive smoking and lung cancer and concluded that the existing evidence is highly suggestive that long-term exposure to environmental tobacco smoke increases the risk of lung cancer. Summarising the available data, they estimated that the excess risk was ~30%. The excess risk rose with increasing exposure, reaching ~70% among heavily exposed non-smokers. Wald *et al.* (1986) also calculated a relative risk of 1.35 for lung cancer among non-smokers living with smokers by pooling the results of 10 case control studies and three prospective studies and concluded that breathing other people's tobacco smoke is a cause of lung cancer. Compared to the 13 studies included by Wald *et al.* (1986) the present study included the largest series of never smoking lung cancer cases (199 cases). Results of the present study would add more evidence on passive smoking as a risk factor and they would contribute towards part of the explanation for the high incidence of lung cancer in never smoking women in Hong Kong.

With regard to the possibility of bias through the misclassification of current and ex-smokers as lifelong non-smokers, Wald *et al.* (1986) stated that the extent of misclassification bias was influenced by the proportions of men and women in the population who had smoked at some time and the greater the proportions (of women in particular), the greater the bias. By choosing the high proportions of 50% of smokers in women and 70% in men and a low observed relative risk of 1.35, they concluded that the misclassification bias was unlikely to account for all the association between lung cancer and passive smoking. In Hong Kong, the proportion of smokers in men was 32.8% and in women 4.1% (Hong Kong Census and Statistics Department, 1985). These figures, particularly in women, were much lower than the figures used by Wald *et al.* (1986). Also, the observed RR was higher in the present study. Thus the extent of influence by misclassification bias would be much less and could not account for the relatively high RR in the present study.

Furthermore, a comparison for adenocarcinoma on the RR due to active smoking (1.87) and that due to passive smoking (2.12) seemed to suggest that the risk for passive smoking was quite similar to that for active smoking for this particular cell type. This was not the case for all other cell types in which active smoking posed much higher risks than passive smoking. The apparently greater risk of adenocarcinoma than of other cell types from passive smoking conflicts with findings in other studies and this may be a feature of small numbers. However, Peto and Doll (1986) in their recent editorial on passive smoking stated that the observed risk need not necessarily be the same in all countries as type of tobacco, past changes in smoking habits, and the extent of passive exposure both at home and elsewhere may all differ substantially between different countries. In places like Hong Kong where people lived in more over-crowded conditions with poor ventilation, passive exposure may be heavier resulting in a higher RR. Moreover, Wynder and Goodman (1983) noted that the predominant cell type of lung cancer in non-smokers is adenocarcinoma and postulated that passive inhalation may primarily increase the risk for adenocarcinoma because side-stream smoke, which contains many gaesous components, can reach the deeper parts of the lung more readily than can mainstream smoke with more particulates. Together with the findings by Lam (1985) on peripheral adenocarcinoma, our results do offer some support for Wynder and Goodman's postulate that passive smoking may be a risk factor particularly for adenocarcinoma. At the very least, reviews of passive smoking and lung cancer can no longer suggest that the results in Hong Kong fail to support the existence of a real relationship.

In conclusion, however, we note that 25.2% (53/210) of our patients with adenocarcinoma were neither smokers themselves nor passive smokers due to smoking husbands. Although smoking and passive smoking may account partly for the high incidence of adenocarcinoma, exposure to other factors should be further examined to elucidate the aetiology of lung cancer, particularly the high incidence of adenocarcinoma in this population.

We are most grateful to the International Development Research Centre and University of Hong Kong for their very generous support in providing the research grants to this project and to Dr D.W. Han for his continuous support and advice. We wish to thank the medical superintendents of Grantham Hospital, Kowloon Hospital, Kwong Wah Hospital, Nam Long Hospital, Ruttonjee Sanatorium and United Christian Hospital for their permission to interview the patients and the staff involved, particularly the pathologists for their co-operation; to Mrs J. Cheang, Mrs J. Wong, Miss S.C. Wong, Miss Connie Wu, Miss C.W. Yip and Miss Rita Lo for interviewing and other research assistance; to Miss Agnes Chow and Mrs T. Lam for their secretarial assistance and to all the interviewees for their co-operation and participation. Finally, we are indebted to Dr M.J. Colbourne for his comments on the technical report submitted to I.D.R.C. We are particularly grateful to Mr Richard Peto and Sir Richard Doll for reading and commenting on the report and for their encouragement.

### References

BLOT, W.J. & FRAUMENI, J.F. (1986). Passive smoking and lung cancer. *J. Natl Cancer Inst.*, **77**, 993.

BRESLOW, N.E. & DAY, N.E. (1980). *The analysis of case control studies*. International Agency for Research on Cancer: Lyon.

CHAN, W.C., COLBOURNE, M.J., FUNG, S.C. & HO, H.C. (1979). Bronchial cancer in Hong Kong 1976–77. *Br. J. Cancer*, **39**, 182.

CHAN, W.C. & FUNG, S.C. (1982). Lung cancer in non-smokers in Hong Kong. In *Cancer campaign*, **Vol. 6.** Cancer epidemiology, Grundmann, E. (ed) p. 199. Fischer Verlag: Stuttgart and New York.

DIRECTOR OF MEDICAL AND HEALTH SERVICES OF HONG KONG (1986). *1985–1986 Departmental Report*. Government Printer: Hong Kong.

HIRAYAMA, T. (1981). Non-smoking wives of heavy smokers have a higher risk of lung cancer: A study from Japan. *Br. Med. J.*, **282**, 183.

HONG KONG CENSUS & STATISTICS DEPARTMENT (1985). *Special Topics Report III*, Social Data Collected by the General Household Survey. Government Printer: Hong Kong.

IARC WORKING GROUP (1985). *IARC Monographs on the Evaluation of the Carcinogenic Risks of Chemicals to Humans: Tobacco Smoking*. **Vol. 38.** International Agency for Research on Cancer: Lyon.

KOO, L.C., HO, J.H.C. & SAW, D. (1984). Is passive smoking an added risk factor for lung cancer in Chinese women? *J. Exp. Clin. Cancer Res.*, **3**, 3.

KOO, L.C., HO, J.H.C. & LEE, N. (1985). An analysis of some risk factors for lung cancer in Hong Kong. *Int. J. Cancer*, **35**, 149.

KUNG, I.T.M., SO, K.F. & LAM, T.H. (1984). Lung cancer in Hong Kong Chinese: Mortality and histological types, 1973–1982. *Br. J. Cancer*, **50**, 381.

LAM, W.K. (1985). *A clinical and epidemiological study of carcinoma of lung in Hong Kong*. M.D. Thesis, University of Hong Kong: Hong Kong.

LAM, W.K., SO, S.Y. & YU, D.Y.C. (1983). Clinical features of bronchogenic carcinoma in Hong Kong: Review of 480 patients. *Cancer*, **52**, 369.

MEDICAL RESEARCH COUNCIL'S COMMITTEE ON RESEARCH INTO CHRONIC BRONCHITIS (1966). Questionnaire on Respiratory Symptoms, UK.

PETO, J. & DOLL, R. (1986). Passive smoking. *Br. J. Cancer*, **54**, 381. *(editorial)*

TRICHOPOULOS, D., KALANDIDI, A., SPARROS, L. & MACMAHON, B. (1981). Lung cancer and passive smoking. *Int. J. Cancer*, **27**, 1.

WALD, N.J., NANCHAHAL, K., THOMPSON, S.G. & CUCKLE, H.S. (1986). Does breathing other people's tobacco smoke cause lung cancer? *Br. Med. J.*, **293**, 1217.

WATERHOUSE, J., MUIR, C., SHANMUGARATNAM, K. & POWELL, I. (eds) (1982). *Cancer Incidence in Five Continents*, **Vol. IV.** IARC Scientific Publications No. 42. International Agency for Research on Cancer: Lyon.

WYNDER, E.L. & GOODMAN, M.T. (1983). Smoking and lung cancer: Some unresolved issues. *Epidemiol. Rev.*, **5**, 177.